# Pragmatic Effect of Lower Limb McKenzie in Grade One Osteoarthritis: A Novel Case Report

**DOI:** 10.7759/cureus.29945

**Published:** 2022-10-05

**Authors:** Samiksha Khemani, Saloni Shah, Shrikant Mhase, Sabih N Khan, Akshay M Nimje, Manoj S Patil

**Affiliations:** 1 Rehabilitation, Mahatma Gandhi Mission (MGM) School of Physiotherapy, Aurangabad, IND; 2 Department of Community Physiotherapy, Mahatma Gandhi Mission (MGM) School of Physiotherapy, Aurangabad, IND; 3 Department of Cardiorespiratory Physiotherapy, Mahatma Gandhi Mission (MGM) School of Physiotherapy, Aurangabad, IND; 4 Research, NKP Salve Institute of Medical Sciences and Research Center, Nagpur, IND; 5 Research and Development, Jawaharlal Nehru Medical College, Datta Meghe Institute of Medical Sciences, Wardha, IND

**Keywords:** functional incompetency, range of motion, mckenzie technique, manual therapy, knee osteoarthritis

## Abstract

Knee osteoarthritis (OA) is a usual disorder depicted as discomfort and loss of functional performance, including decreased proprioceptive acuity. Symptoms of this condition include stiffness, pain, swelling, joint disproportion, functional incompetency, and muscle atrophy, which may reduce the well-being of the patient. This report highlights the case of a 41-year-old female patient who complained of right knee pain, which was persisting for a month, and was treated with the McKenzie Technique (MT) along with electrotherapy modalities. Following 12 days protocol, there was a substantial reduction in pain, improvement in functional ability and knee muscle strength, and reduced walking time. Various other physiotherapy approaches and techniques were inculcated in the management of OA, which involved a soft tissue approach, therapeutic training, and manual method. Hence, this case report highlights the pragmatic effect of lower limb MT in grade 1 knee OA patients, which may improve the patient’s well-being.

## Introduction

Osteoarthritis (OA) or degenerative arthritis occurs when the joints' cartilage is irritated, damaged, and eventually lost. Being one of the most prevalent forms of arthritis, its frequency of occurrence increases with age [1]. Mobility and movements within the joints cause the release of synovial fluid from the synovial membrane, which nourishes different avascular parts in the knee joint. Although, because of its regular usage and significant stress, this joint is the most prevalent site for painful diseases, including knee OA where the biomechanical qualities of articular cartilage are altered [2-4]. Knee OA is a usual disorder depicted as discomfort and loss of functional performance, including decreased proprioceptive acuity [5]. Furthermore, articular cartilage degradation and loss are observed during osteophyte growth, synovial membrane inflammation, and hypochondriac bone disintegration [6,7]. Symptoms of this condition include stiffness, pain, swelling, joint disproportion, functional incompetency, and muscle atrophy, which may reduce the well-being of the patient [7].

The McKenzie system of mechanical diagnosis and therapy (MDT) is one of the most potential exercise-based interventions that can be employed to treat knee OA in which the "derangement" classification of the MDT is the most common and extensively researched subcategory. This classification which correlates with "directional preference," has been identified for all joints and was related to a quick reaction to specific end-range movements. Directional preferences in the knee can be either flexion or extension type. Based on a directional-preference assessment for patients with knee OA, this diagnosis and therapy may provide a more targeted exercise prescription [8]. Manual therapists may stimulate a variety of specific and other non-specific changes in the patient's body by treating the musculoskeletal structures of the body, which include joints, soft tissues, and nerves, which can result in an appreciable increment in the patient's functioning in the long run [9,10].

Since there is no curative therapy for treating knee OA, total knee replacement (TKR) is chosen based on the seriousness of the patient's condition. However, only about 19% of the patients are satisfied with these surgeries. Moreover, due to the heavy economic burden on healthcare, it is important to explore other options [8]. This case study's objective was to assess the McKenzie Technique's (MT) short- and long-term efficiency in treating knee OA patients with regard to pain management, enhancing knee mobility and functional performance with ultimately improving the patient’s well-being.

## Case presentation

A 41-year-old female presented to the general outpatient department with the primary complaint of pain in the right knee, which was gradual in onset, continuous in nature, and aggravated on activity persisting for a month. She consulted an orthopedic surgeon who prescribed her analgesics to relieve pain; the duration of the prescribed course was five days, and also advised physiotherapy treatment. But the patient only took medications without physical therapy; still, the pain was constant, and her condition worsened due to bearing heavy weight, so she ultimately decided to undergo physiotherapy treatment. On observation, the patient exhibited distinct postural deviation, which consisted of knee hyperextension (genu recurvatum), and foot eversion (foot turned out).

On physical examination, all vital parameters of the patient, including body temperature (98.6 F), heart rate (79 beats per minute), blood oxygen saturation level (97%), and blood pressure (140/90 mmHg), were found to be within normal ranges. The pain intensity recorded on the numerical pain rating scale (NPRS) was eight on activity, three at rest, and aggravated during stair climbing and descending. On palpation, the medial and lateral aspects of the right knee revealed grade 2 tenderness with a positive crepitus sign. On assessment, the range of motion (ROM) for knee flexion and extension was restricted and painful. Muscle strength was grade 3+ in the knee flexors, three in the knee extensors and hip flexors, and four in the hip extensors of the right side.

**Figure 1:**
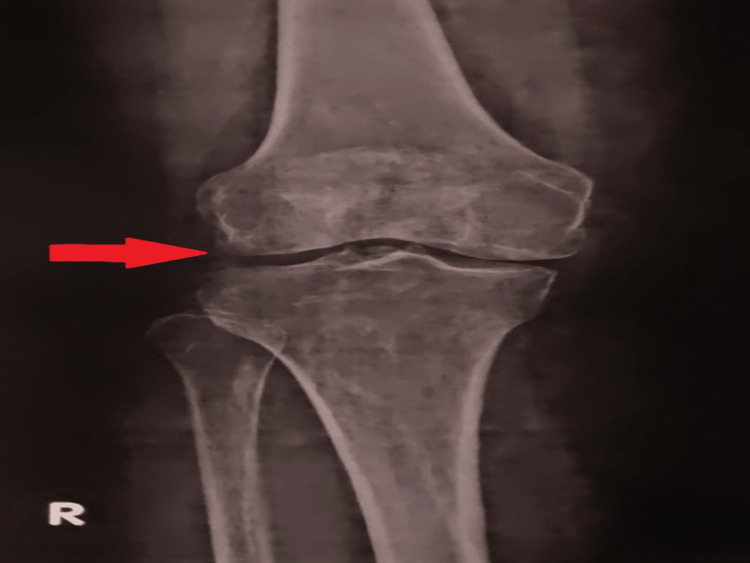
X-ray showing reduced joint space and osteophyte formation.

In order to rule out the severity of arthritis, radiographic investigations were carried out in which the patient underwent an X-ray of the right knee, which revealed moderate joint space reduction in the right knee with the possible formation of osteophytes indicating grade 1 OA [11] (Figure 1).

A thorough history of the diagnostic process of the MDT system classification was used to determine which intervention needed to be put into practice. In this case, each knee movement was evaluated separately, and the effects on the control activity (for instance, stair ascending) were assessed in turn with repeated end-range motions in various loading postures. The MDT derangement group was given precise end-range exercises in the direction the patient had indicated. For instance, if the patient's preferred direction was determined to be knee extension, end-range knee extension movements were given to them depending on which loading strategy they responded to the best [8].

Before commencing Physiotherapeutic Intervention (PI), the patient was educated about her condition and was briefed about the intervention protocol. The patient was made aware that her medical records will be kept confidential, and written informed consent was received. The PI began with electrotherapy modalities (EM) consisting of cryotherapy, which reduced swelling, followed by Transcutaneous Electrical Nerve Stimulation (TENS) in combination with Interferential Therapy (IFT) over the medial and lateral aspects of the knee was given. Each modality was given for 10 minutes which was followed by MT.

**Figure 2:**
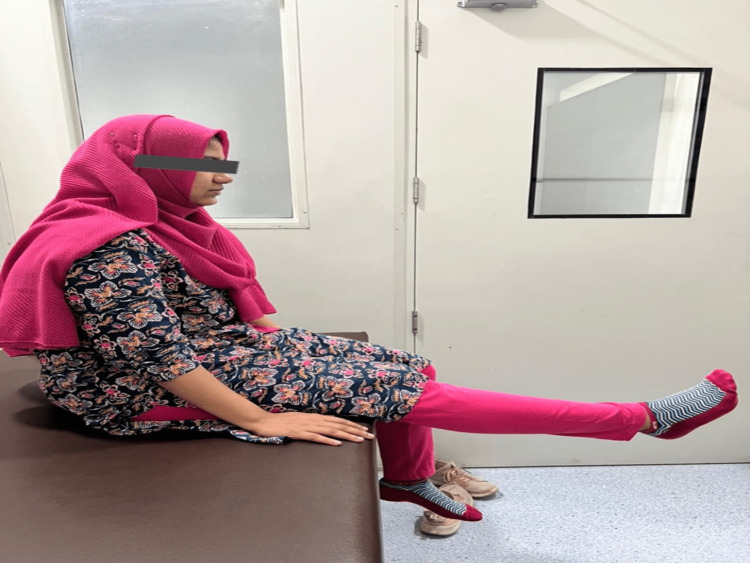
Active knee extension in sitting

Based on the MDT, the directional preference for the patient was of extension type. In a sitting position with minimal loading, the patient was made to sit with the knee at 90° on a stool or chair, after which the knee was extended to the farthest extent possible, held for three to five seconds, and then brought back to the beginning position (Figure 2) [8].

**Figure 3:**
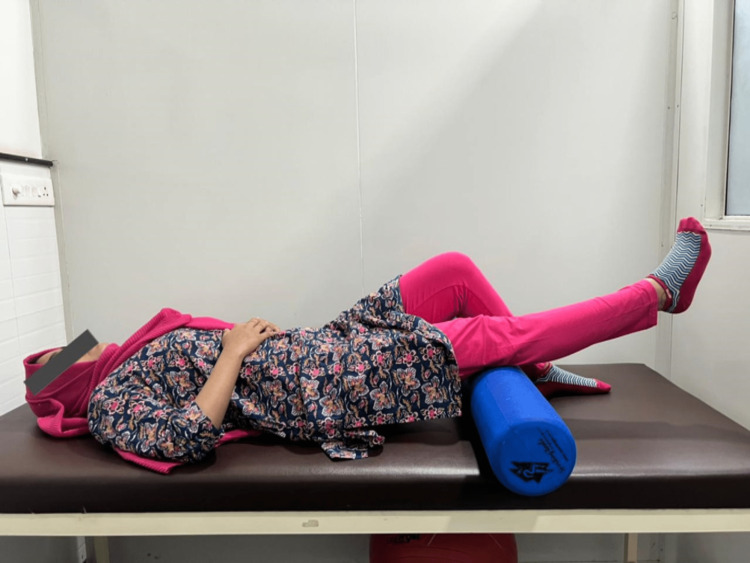
Active knee extension in lying.

In addition, with a rolled towel positioned just proximal to the knee, the knee extension was actively performed while the patient was supine. The patient voluntarily extended the knee up to the pain-free range possible, held the position for three to five seconds, and then gradually returned to the beginning position (Figure 3) [8].

**Figure 4:**
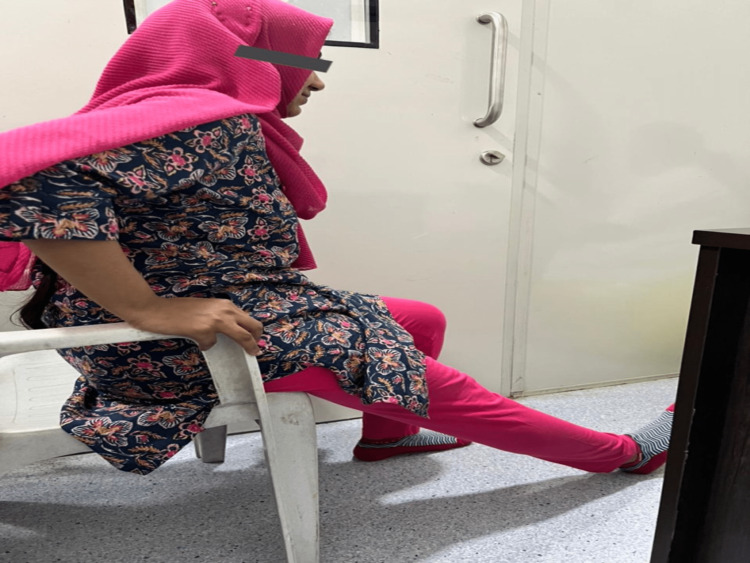
Knee extension in sitting

Further, the patient was told to lean back on the edge of the chair, support the extended knee on the floor, and be allowed to sag into further extension. The hands provide pressure on the knee to allow it to extend further. The patient held this position for three to five seconds before shifting back to the initial position (Figure 4) [8].

The frequency of repetition of MT exercises was 10 repetitions every 2 to 3 hours, which varied according to the condition and capabilities of the patient and was continued for two weeks under the supervision of the therapist [8].

Further, strengthening exercises performed were static quadriceps, static hamstrings, vastus medialis oblique strengthening, and straight leg raise consisting of 1 set of 10 repetitions with a 10-seconds contraction hold. Along with this, sit-to-stand, wall slides, step-downs with the unaffected leg leading, and step-ups with the affected leg leading were performed, consisting of 1 set of 10 repetitions.

At the start of PI, patient-reported pain intensity was eight on NPRS, which was reduced to one at the end of the session. The patient, in total, completed 12 days of the session, after which a remarkable increase in ROM and muscle strength was observed, as shown in Table 1.

**Table 1:** Outcomes describing pre- and post-treatment values. NPRS: Numerical Pain Rating Scale, ROM: Range of Motion

Outcome	Pre-treatment values	Post-treatment values
Pain	8 on the NPRS	1 on the NPRS
ROM of the affected right side in degrees by using a goniometer		
Flexion	0- 85	0-100
Extension	85-0	100-0
Muscle strength of right side in grades by manual muscle testing		
knee flexors	3+	4
Knee extensors	3	4
Hip flexors	3	4
Hip extensors	4	4
Tenderness in grades		
Medial side of the knee	2	1
Lateral side of the knee	2	1

## Discussion

This case report highlights the pragmatic effect of lower limb MT in grade 1 knee OA patients and emphasizes its appreciable impact in reducing knee pain, as well as enhancing ROM, muscle strength, and performance post-therapy. In 1981, the MT or Mechanical Diagnosis & Treatment (MDT) was established by physiotherapist Robin McKenzie, and it is a system for diagnosing and treating disorders of the spine and extremities. The distinctive feature involves an emphasis on patient empowerment and self-medication. MDT subdivides patient complaints according to the patient's clinical presentation rather than categorizing them anatomically. The reliability of MDT classification has been confirmed by several studies [12].

When the impact of Mulligan Mobilization with Movement Technique in conjunction with conventional PI and MT with conventional PI were compared in knee OA patients, it was found that using MT and mobilization with a movement approach resulted in positive improvements in the functional end-result and knee ROM. The MT was also found efficacious in ameliorating the extensor strength of the knee [4]. Similar findings had been sought in a study that described that MT produced very noteworthy outcomes in terms of lowering pain, dysfunction, and enhanced functional movements, as well as tolerance [13]. For acute low back pain, the MT is highly efficacious in comparison to passive management and reduces the magnitude of pain [14].

Along with MT, EM also played an essential role in decreasing pain intensity. The utmost objective of the IFT is to lessen pain and fasten patient healing as part of a physical therapy or rehabilitation program. This therapy has fewer adverse effects as it is a non-invasive, drug-free treatment. It infiltrates into the body to a deeper level and extends to the origin of pain with comfort [15]. To evaluate IFT's efficacy in treating knee OA, a meta-analysis was carried out, which comprised four research and found that IFT paired with therapeutic exercise helps patients with knee OA feel less pain [16]. In order to treat knee OA, the impact of TENS and IFT in conjunction with exercise was examined in a randomized experiment in which WOMAC OA index and pain intensity were used to measure the final approach, and within a four-week period, with no additional effects of IFT or TENS on pain and function compared to the therapeutic exercise alone, there were overall improvements that were noticeable therefore, exercise was crucial in the therapy of OA patients [17]. Whereas, in the present study, the patient has shown a good prognosis in accordance with IFT.

To acquire muscle strength, two muscles are involved quadriceps and hamstrings, which provide stabilization to the knee joint and increase joint compression by contracting [18]. The different approach, like resistance exercises are essential in the management of mild and moderate knee OA, which provides numerous benefits, such as relieving symptoms, less dysfunction, enhanced functional capacity, and overall well-being [19]. In a comprehensive review and meta-analysis of randomized controlled trials, one of the key goals and the focal point of a good exercise program for individuals with knee OA was found to be quadriceps strengthening [20]. A meta-analysis also established that strengthening exercises along with or without weight bearing was beneficial in lowering pain. Hence, it is crucial to recognize the eminent technique to enhance the strength of the quadriceps muscle for a better prognosis [21].

To keep the knee stable and avert overstrain, it's crucial to have strength of quadriceps proportional to hamstrings. It is, therefore, more important in therapy to strengthen the quadriceps and hamstrings. Resistance training and MT were more efficacious in enhancing functional performance, including knee extension strength, range of motion, and climbing of staircase. By performing thrice a week, functional exercises, along with exercise of ankle weight-strengthening such as step-ups, squats, hip abduction or adduction, decreases the uneasiness, improves leg capability, enhances climbing and ultimately improving functional performance of the patient [22].

## Conclusions

This case report concludes that McKenzie Technique can contribute effectively to combating knee OA by managing pain and enhancing functional performance. Therefore, it can be suggested to apply it in combination with conventional PI. Additional studies are required to support these conclusions and evaluate the impact of the MT as an analgesic effect on the patient with or without medication management by comparing the effectiveness of the MT with that of other therapeutic techniques and methodologies, both in the short and long term.
